# A Sensitive, One-Way Sequential Sieving Method to Isolate Helminths’ Eggs and Protozoal Oocysts from Lettuce for Genetic Identification

**DOI:** 10.3390/pathogens9080624

**Published:** 2020-07-31

**Authors:** Annina R. Guggisberg, Cristian A. Alvarez Rojas, Philipp A. Kronenberg, Nadia Miranda, Peter Deplazes

**Affiliations:** Institute of Parasitology, Vetsuisse and Medical Faculty, University of Zürich, 8057 Zürich, Switzerland; annina.guggisberg@uzh.ch (A.R.G.); philipp.kronenberg2@uzh.ch (P.A.K.); nadiamiranda@bluewin.ch (N.M.)

**Keywords:** foodborne parasites, *Echinococcus multilocularis*, *Toxoplasma gondii*, *Toxocara* spp.

## Abstract

Different helminths and protozoa are transmitted to humans by oral uptake of environmentally resistant parasite stages after hand-to-mouth contact or by contaminated food and water. The aim of this study was to develop and validate a method for the simultaneous detection of parasite stages from fresh produce (lettuce) by a one-way isolation test kit followed by genetic identification (PCR, sequencing). Three sentinel zoonotic agents (eggs of *Toxocara canis*, *Echinococcus multilocularis* and oocysts of *Toxoplasma gondii*) were used to investigate the practicability and sensitivity of the method. The detection limits (100% positive results) in the recovery experiments were four *Toxocara* eggs, two *E. multilocularis* eggs and 18 *T. gondii* oocysts (in 4/5 replicates). In a field study, helminth DNA was detected in 14 of 157 lettuce samples including *Hydatigera taeniaeformis* (Syn. *Taenia taeniaeformis*) (four samples), *T*. *polyacantha* (three), *T*. *martis* (one), *E*. *multilocularis* (two) and *Toxocara cati* (four). *Toxoplasma gondii* was detected in six of 100 samples. In vivo testing in mice resulted in metacestode growth in all animals injected with 40–60 *E. multilocularis* eggs, while infection rates were 20–40% with 2–20 eggs. The developed diagnostic strategy is highly sensitive for the isolation and genetic characterisation of a broad range of parasite stages from lettuce, whereas the sensitivity of the viability tests needs further improvement.

## 1. Introduction

A plethora of helminths and protozoa are transmitted to humans by oral uptake of parasite stages which can survive harsh conditions for long periods of time in the environment. Worldwide, more than a billion people are infected with soil-transmitted human intestinal helminths (STHs), including roundworms, hookworms, and whipworms [1,2]. Furthermore, humans are hosts of a range of protozoa with environmentally resistant stages such as cysts or oocysts of *Giardia lamblia*, *Cryptosporidium* spp., *Entamoeba*, *Cyclospora* and *Toxoplasma* [3,4]. The intestinal parasites occur mainly in tropical and subtropical regions, with insufficient access to clean water and inadequate or absent sanitation. Based on such epidemiological conditions, several studies depicting activities to improve water management, sanitation, and hygiene (WASH) have documented a reduction in the incidence of human soil-transmitted parasites [5]. Furthermore, a range of zoonotic parasites is transmitted to humans via environmental stages excreted in animal faeces. Intestinal zoonotic helminths and protozoa of free-roaming canids including domestic dogs and foxes as well as those from free-roaming domestic cats play a major role in contaminating the environment, including recreational areas, private kitchen gardens and agricultural areas, with resistant parasite stages [6,7,8,9]. Especially, the uncontrolled growth of stray dog populations in South America, Africa, large parts of Asia and Southern Europe represent an emerging “One Health” problem as a source of zoonoses [10]. Furthermore, increasing populations of red foxes, coyotes and raccoons in agricultural and rural areas have augmented the risk of transmission for zoonotic parasites in Europe and North America [6,11,12].

The transmission of environmentally resistant parasite stages to humans is complex and can be attributed to contaminated food, water, soil and human behaviour such as hand-to-mouth contact [13]. Hands can be contaminated by close contact with soil, plants, water and also through direct contact with definitive hosts of certain parasites. For example, dog ownership has been identified as a risk factor for patients with alveolar echinococcosis, caused by the tapeworm *Echinococcus multilocularis* [14]. On the other hand, the association between toxoplasmosis and close contact with cats is unclear [15,16,17]. While eggs of the nematode *Toxocara canis* are often found on the coat of infected dogs or foxes, they are rarely fully embryonated and therefore not immediately infectious [7,18]. In 2015, a ranking based on a multicriteria assessment (MCA) approach (including quantity, severity, global distribution and potential for short-term emergence) of the 24 most important parasites potentially transmitted by food, was published by the WHO/FAO [19]. The soil-transmitted helminths *Ascaris* spp. and *Trichuris trichiura* were ranked 9th and 15th, respectively. *Echinococcus granulosus sensu lato*, *E*. *multilocularis* and *Toxoplasma gondii* were top-ranked, and *Toxocara* spp. were placed in the bottom quarter of the list. According to the authors, fresh produce was considered as the primary vehicle for food transmission of *Echinococcus* and *Toxocara* eggs while meat is considered to be the primary source of infection for *T*. *gondii*. The link between infection/disease with a foodborne parasite and its origin (source attribution) is difficult to establish for many of these pathogens, mostly due to the long incubation time (i.e. alveolar and cystic echinococcosis caused by *E*. *multilocularis* and *E*. *granulosus s.l.*, respectively). Moreover, food is not the only source for parasites transmitted to humans, and so far quantitative estimations of the role of food in the transmission could not be established [20]. Overall, the role of food in the transmission of *Echinococcus* spp. has recently and controversially been discussed [21,22,23]. Therefore, the proper documentation of food as a vehicle of parasite transmission is paramount to elucidate its relevance for human infection.

Several studies, based on microscopic detection of parasite stages in fresh produce for human consumption, have reported different rates of contamination worldwide (summarised in [21]). However, microscopy has limited specificity and sensitivity for the detection of eggs/(oo)cysts in fresh produce (and environmental samples). For example, there are difficulties concentrating and separating eggs and (oo)cysts from debris and soil, especially when a low number of environmental stages is present in large quantities of the analysed material. Therefore, a molecular approach is crucial for the correct identification of parasite stages and also for improving the sensitivity of the method. A variety of diagnostic procedures have been developed/validated in the last decades, mainly focusing on the monospecific detection of pathogens, with high potential to be used for food investigations [20]. However, a strategy with the potential of detecting simultaneously the full range of foodborne parasitic pathogens is still missing. It is important to consider that detection of parasite DNA does not imply that the eggs/(oo)cysts present in food sources are viable. It is possible that these environmental stages can “mummify”, becoming non-viable but preserving DNA for decades. Some studies have addressed this issue, e.g. different methods have been developed for assessing the viability of oocysts of *T. gondii* using in vivo and in vitro methods (reviewed in [24]). For *E*. *multilocularis* eggs, it is possible to assess their viability with an in vivo method using subcutaneous injection of sodium hypochlorite resistant oncospheres [25]. This method was further validated in the present study, while there is no method available to estimate the viability of eggs of *E*. *granulosus s.l.* or *Taenia* spp. [21]. In the case of *Toxocara* spp., the viability of eggs can be confirmed through microscopic detection of moving larvae.

The aim of this study was to develop and validate a practical and straightforward method for the isolation of environmentally resistant parasite stages from fresh produce (lettuce) and minimizing laboratory DNA contamination by a one-way system. Furthermore, the practicability and sensitivity of the assay were validated by using parasite stages of three sentinel zoonotic agents with different sizes: large embryonated eggs of *Toxocara* spp. (70–90 µm), medium-sized viable eggs of *E. multilocularis* (30 to 40 μm) and small sporulated oocysts of *T. gondii* (11 × 13 μm).

## 2. Results

### 2.1. Recovery of Parasites in Spiked Lettuce Samples

Table 1 shows the result for the detection of *Toxocara* and taeniid eggs after spiking 300 g (g) of lettuce and washing the vegetables with the sieving system presented in this study. All replicates spiked with *Toxocara* eggs were PCR positive when testing the 40 µm filter fraction. All replicates spiked with 20, and four taeniid eggs were positive for *E. multilocularis* in PCR (21 µm filter), while 2/5 and 3/5 were PCR positive for *Taenia*, respectively. No DNA amplification in both PCRs was observed in three control lettuce samples. The second half of the sediment from the 40 µm filters was examined in the microscope after flotation with ZnCl_2_. *Toxocara* eggs were visible in all five replicates from lettuce spiked with 20 eggs and in 2/5 replicates after spiking with four embryonated *Toxocara* eggs. The presence of taeniid eggs was not systematically investigated by microscopy, due to the high content of co-purified particles and for biosafety reasons. However, during pilot studies in the current investigation, taeniid eggs were found by microscopic examination in several cases after a flotation step (data not shown).

Table 2 shows the results for the detection of *E. multilocularis* eggs and *T. gondii* oocysts after spiking 300 g of lettuce and washing as described in Material and Methods. All five replicates were positive for *E*. *multilocularis* in lettuce spiked with 2–120 eggs. The second half of the sediment of samples spiked with 20, 40 and 120 eggs was used for in vivo viability testing, and results are shown in a further section of the study. From the second half of the 21 µm filter from samples spiked with two and 10 eggs, a PCR was performed showing a positive result for *E*. *multilocularis* in all five replicates spiked with 10 eggs and in 1/5 replicates when lettuce was spiked with two eggs (Table 2). Recovery of *T*. *gondii* after spiking revealed positive PCRs in the flow-through of all five replicates spiked with 86 oocysts and in 4/5 replicates after spiking lettuce with 18 sporulated oocysts (Table 2). The three negative control lettuce samples were PCR-negative for all parasite targets. Furthermore, triplicates spiked with 779 µL and 130 µL egg-free supernatant of the *E. multilocularis* spiking suspension were all PCR negative (Table 2).

### 2.2. Detection of Taeniids, Toxocara spp. and Toxoplasma gondii in Lettuce Purchased in Farmer Markets and Supermarkets in Zürich

The sieving system was initially tested with a sample weight of 300 g of lettuce leaves used for the spiking experiments. The sample weight was scaled up to 900 g for the field study aiming to analyse the highest number possible of lettuce heads. Furthermore, according to the amount of dirt present in the lettuce, the diagnostic unit was further scaled up to 1200, 1500 and 1800 g. Of the 157 units investigated here, 119 consisted of 900 g, 21 of 1200 g, 14 of 1500 g and 3 of 1800 g. We estimated that one person could process five diagnostic units of 900 g in one day (8 h) without considering the time spent purchasing the vegetables and the molecular/microscopic diagnostic and viability analyses. According to the weight of the diagnostic units, we found 11 positives in the 119 units weighing 900 g, and 1/21 (1200 g), 2/14 (1500 g) and 0/3 (1800 g) in the other units.

The results of the detection of eggs of *Taenia* spp., *E. multilocularis* and *T. cati* in 157 lettuce diagnostic units are summarised in Table 3. Taeniids were found in 10 samples and *T. cati* in four. The taeniid species identified included *Hydatigera taeniaeformis* (Syn. *T*. *taeniaeformis*) (in four diagnostic units), *T*. *polyacantha* (three), *T*. *martis* (one) and *E*. *multilocularis* (two). From the 120 diagnostic units analysed from farmer markets, 10 were positive for taeniids and three for *Toxocara cati*. In the diagnostic units originating from supermarkets (n = 37), one was positive for *T*. *cati*. There was no statistically significant difference between the contamination of samples collected in summer and autumn (*p* = 0.94) and between samples from the farmer markets and the supermarkets (*p* = 0.13). In lettuce purchased in supermarkets, no taeniid eggs were identified by PCR, but one sample tested positive for *T*. *cati*.

Six positive PCRs were found in the 100 diagnostic units investigated for *T*. *gondii* (Table 4). There was no statistically significant difference between the contamination of samples collected in summer and autumn (*p* = 0.63) and between samples from the farmer markets and the supermarkets (*p* = 0.59).

### 2.3. Validation of Viability Testing: In Vivo Test for E. multilocularis Eggs and by Microscopy for Toxocara Positive Samples

The in vivo viability of the two egg suspensions used for spiking was investigated. In the mice group (n = 5) inoculated with *E. multilocularis* and *T. polyacantha* eggs, metacestode growth was visible in all five animals inoculated with 40 and in two animals per group inoculated with 10 or two taeniid eggs each (Table 5). In the second part of the experiment, mice groups inoculated with 60, 20 and 10 sodium hypochlorite resistant (SHR) *E. multilocularis* eggs developed subcutaneous metacestode growth in five, two and one animal, respectively (Table 5). *E*. *multilocularis* was confirmed in the lesions using PCR in all cases in both experiments.

After spiking samples with the *E. multilocularis* and *T*. *polyacantha* egg suspension, the second half of the 21 µm sediment was inoculated to mice, yielding metacestode growth in only one sample spiked with four eggs (Table 5). Lettuce spiked with *E*. *multilocularis* eggs only produced metacestode growth in 1/5 mice in samples spiked with 120 or 20 eggs each (Table 5). *E. multilocularis* was confirmed by PCR on cyst material in all three cases.

The in vivo testing of taeniid eggs isolated from the field study was investigated with the second half of the sediments collected on the 21 µm filter from diagnostic units which were PCR positive for *E*. *multilocularis* (Table 3). No metacestode growth was observed after 12 weeks. All four samples positive for *Toxocara* DNA were examined under the microscope, but no eggs were detected.

## 3. Discussion

In the present study, we established a practical strategy for the detection of environmental parasite stages (eggs, (oo)cysts) in large quantities of lettuce. The sieving method was originally used for the detection of taeniids eggs in canine faeces [26,28] overcoming at this time major problems with PCR inhibition after direct DNA isolation from faeces or environmental samples (without commercial DNA isolation kits available) and further adapted for the detection of taeniid eggs in food sources for non-human primates [29]. In the latest publication [29], each diagnostic unit consisted of up to 60 kg of fruits and vegetables requiring 250 L of water to wash and pass through the sieving system. Such volumes are difficult to manage and require a sieving system with large filters (16 cm in diameter) and pipes which have to be used multiple times and washed thoroughly between samples to avoid contamination. In the current study, we aimed to decrease the volume of water per unit investigated. We consider 300 g as the typical amount of salad (lettuce) that a family would consume in a meal. However, the diagnostic strategy proposed can be easily adapted for other vegetable or fruit samples. Similar quantities of vegetables per sample (between 200 and 300 g) were investigated in several epidemiological studies searching for parasite eggs in different countries (see [21]). The strategy presented here involves a sedimentation/centrifugation step of the flow-through for the detection of particles passing the 21 µm sieves, which is the case for most protozoal stages. 

Subsequently, we tested the practicability of the diagnostic strategy with 157 lettuce diagnostic units (between 900 and 1800 g) in a field study. In this process, we upscaled the initial amount of lettuce by a factor of three, as we expected the contamination of food sources to be low in Switzerland. Subsequently, according to the amount of dirt in the lettuce, higher amounts were also investigated per diagnostic unit. The majority of diagnostic units (119) consisted of 900 g, 21 weighing 1200 g, fourteen 1500 g and three 1800 g; parasite stages were found in 11 (9%), one (5%), two (14%) and 0, respectively. We suggest that future investigations could be carried out using only 900 g. Furthermore, the established sieving system is easy to assemble and to apply. With the exception of high-quality filters, there is no need for special and expensive equipment. Re-used polyethylene terephthalate (PET) bottles are a key component of the one-way system and are used a single time to avoid any contamination between samples. The egg isolation using the prepared ‘sieving kit’ can be performed in basic laboratories or even in private kitchens. In fact, as part of a pilot study, sieving kits were given to people who were concerned with the presence of parasite stages in seasonal wild vegetables used for human consumption in Switzerland. These seasonal vegetables were washed and sieved at home, and taeniids were identified in the laboratory in two samples (one *T*. *martis* and one *T*. *crassiceps*, unpublished data). This result shows the reliability and easy-to-perform feature of the system. This also shows that in future investigations community-based investigations could be organised in citizen science projects for the examination of large quantities of food from different sources, surpassing the monitoring capacity and resources that currently exist. The concept of citizen science has provided benefits in different fields such as ecology [30] and health disparity studies [31], for example. However, the second part of the method proposed here, including the molecular detection, requires highly qualified staff and professional lab infrastructure and consumables for DNA isolation and PCR.

The established method is able to identify as few as two embryonated *Toxocara* eggs and one–two *E*. *multilocularis* eggs in samples consisting of 300 g of lettuce. The method reported by Frey et al. [32], which is based on a centrifugation step of the washing water containing 0.1% alconox or glycine followed by a real-time PCR version of the multiplex PCR used in the present study [26], had a detection limit of five taeniid eggs (*T*. *pisiformis*) per 35 g of lettuce. However, the feasibility of the method was not tested with a field study. We privilege the use of a traditional PCR rather than a real-time approach because the methodology proposed here is intended to be implemented in countries where a real-time PCR device might not be easy to acquire due to financial restrictions. The multiplex PCR is also designed for general identification of taeniids after sequencing the PCR products; this poly-specific approach allowed us to identify *T*. *saginata* in some preliminary experiments in one lettuce batch originating from other European countries (unpublished data). Two other recent publications established different methods for the investigation of *Echinococcus* spp. in berry samples. One publication could detect 50 *E. multilocularis* eggs from 250 g of bilberries by washing with 5 L of water containing 0.04% Tween20, sieving and qPCR [33]. The other publication reported that five *E. multilocularis* eggs could be detected in 30 g of berries by multiplex qPCR after washing with 200 mL 1% alconox and centrifugation [34]. Nevertheless, the different matrixes used for recovery experiments needs to be considered making it difficult to compare the results. Other investigations performed in Poland, using overnight sedimentation of washing water (2 L) combined with sieving using a 50 µm filter and flotation with ZnCl_2_, documented a detection limit of 100 *E*. *multilocularis* eggs per 500 g of vegetables using a nested PCR [35]. A field investigation by the same authors described 23% of 103 samples positive to DNA of *E*. *multilocularis* from fruit, vegetables, and mushrooms but no microscopic examination of the samples for assessing the presence of eggs was performed. According to the detection limit reported by the authors, the positive samples were contaminated with at least 100 eggs of *E*. *multilocularis*. This extremely high contamination has been questioned by some authors [21,23]. We expected only few diagnostic units to be contaminated with *E*. *multilocularis* eggs; in fact, we found only 2/157 (1.2%) diagnostic units positive. Federer et al. [29] did not find *E*. *multilocularis*; however, 2 out of 46 (4.3%) vegetable samples from unspecified European countries (including South Europe) were positive to *E*. *granulosus*.

For our detection strategy, we intended to use the same sampling method for the investigation of both helminths and protozoal stages. Therefore, the flow-through of the washing solution free of particles >21 µm was further investigated for protozoal stages. For testing the practicability of this approach, a simple overnight sedimentation step at 4 °C was included, followed by centrifugation of the sediment before DNA extraction. Our established method was able to identify at least 18 *Toxoplasma* oocysts in 4/5 samples of 300 g of lettuce. Such low levels of detection as 10 and ≥100 oocysts using a PCR targeting the B1 gene and the 529 bp fragment respectively have been reported [36]. Hohweyer et al. [37] detected even less than 1 *T*. *gondii* oocyst per g of vegetables (33 oocysts/g in 30 g of basil leaves). A study from Poland established a method for *T*. *gondii* with a detection limit of 100 and 10,000 oocysts for 20 radish and 1 kg of strawberries, respectively, using 2 L of Tween20 water, overnight sedimentation, centrifugation and real-time PCR [38]. We acknowledge that more sophisticated molecular tests, the inclusion of internal amplification inhibition control and the use of PCR targets which are highly repeated in the genome of the parasite could be implemented for the investigation of the flow-through [39]. However, as in the case of taeniids and *Toxocara*, we focused on delivering a method for parasite stage isolation which can readily be used and implemented in different laboratories worldwide and which can perform classical PCR.

In the included field study investigating lettuce purchased in farmer markets and supermarkets of the city of Zürich, we chose taeniids, *Toxocara* spp. and *T*. *gondii* as sentinels of environmental contamination with the faecal matter of canids, cats and other definitive hosts of taeniids like mustelids. We detected two typical fox taeniids, *T. polyacantha* and *E. multilocularis*, specific intestinal cat parasites, *Toxocara cati* and *T. gondii*, further *Hydatigera taeniaeformis* (occurring mainly in cats), and *T. martis*, a species occurring in mustelids and rarely diagnosed as zoonotic agents in humans [40]. Interestingly, a previous study using much larger numbers of vegetables per sample (around 40 salad heads and different fruits) investigated in 2016 in Switzerland detected taeniid eggs in 16% of the samples (including two samples with *E*. *granulosus*) [29]. In the present study, based on much smaller sample weight, we detected 6.4% of samples containing taeniid eggs (including two samples with *E*. *multilocularis*). These two *E. multilocularis* samples were found during autumn. In the cold season (autumn/winter), foxes are more often infected with *E*. *multilocularis* than in the warmer season (spring/summer) [41,42]. *Toxocara cati* was detected in four lettuce samples. Furthermore, DNA of *T. gondii* was amplified and confirmed by sequence analyses in six samples, again indicating contamination of food-producing areas with faeces of free-roaming cats. Prevalence of *T*. *gondii* oocysts in different vegetables has shown dissimilar results in studies from different countries: Canada (0%) [43], US (2.5%) [44], Peru (0.79%) [44], Egypt (4.9%) [45], Saudi Arabia (6.6%) [46]. In 2009, according to the Swiss Federal Statistical Office, there were an estimated 1.38 million cats living in Switzerland of which 72% were estimated to have access to outdoors (cited in [47]). Data from Switzerland showed that the frequency of domestic cats shedding *T*. *gondii* oocysts ranged between 0.4% [48] and 0.6% [49].

It is important to keep in mind that molecular detection of environmental parasite stages in any matrix, including food sources, does not imply that such stages are viable. For *Toxocara* spp. viability can be confirmed by observing moving larvae (embryonated eggs). All four positive samples for *Toxocara* spp. by PCR were examined by microscopy, but it was not possible to observe either embryonated or unembryonated eggs. This is most likely to be due to the low number of eggs present and the low sensitivity of microscopy in such samples caused by the presence of many other particles of similar size. In the case of taeniid eggs, the assessment of their maturity/viability has been achieved by exposing them to sodium hypochlorite and counting the number of oncospheres. However, it has been noted that the SHR test is not reliable to assess the viability of long-time preserved *T. hydatigena* eggs (Deplazes and Eckert, 1988) or heat-exposed eggs of *E*. *multilocularis* [25] or *E. granulosus* [50]. In the case of *E*. *multilocularis,* it is possible to assess the viability of the eggs with an in vivo method as used in this study, which is based in the subcutaneous injection of sodium hypochlorite resistant oncospheres to mice [25]. None of the two PCR positive samples for *E*. *multilocularis*, from the field study, injected to mice led to metacestode growth. A possible explanation for this is that a low amount of *E. multilocularis* eggs was present in the sample which did not result in metacestode growth or simply because these eggs were not viable. A previous experimental study showed that at least 20 SHR oncospheres are needed to be injected to produce regular metacestode growth [25]. In the present study, again, only 40 and 60 eggs resulted in consistent metacestode growth, while lower numbers between 2 and 20 eggs caused only infections in 20–40% of the mice. Therefore, the in vivo viability test established [25] is of value for experimental studies with large numbers of eggs but not suitable for field samples with probably very low numbers of viable eggs.

Documenting the transmission of parasites in food is not straightforward because of the lack of standardised and validated methods for this purpose. In this regard, reference laboratories should play a fundamental role in harmonising diagnostic procedures. We present here an easily applicable method which can be readily implemented to detect a broad range of infective parasite stages which can be transmitted by food.

## 4. Materials and Methods

### 4.1. Establishment and Validation of a Method for the Detection of Helminth and Protozoal Environmental Stages in Lettuce

#### 4.1.1. Parasites Eggs and Oocysts

Taeniid eggs were collected at necropsy of foxes originated from the Zürich area during the official hunting season in Switzerland. Intestinal helminths were isolated using the sedimentation and counting method [51]. Intestines of foxes infected with taeniids were selected for isolation of eggs from faeces as previously established [28]. Confirmation of species of the isolated taeniid eggs was achieved by the use of a multiplex PCR [26]. Eggs were stored at 4 °C in phosphate-buffered saline (PBS) with 100 IU of penicillin and 100 μg of streptomycin (Thermo Fisher Scientific, Waltham, MA, USA). The number of eggs was determined under the microscope in a McMaster chamber [52]. Eggs were subjected to a sodium hypochlorite resistance (SHR) test as previously described to establish the number of eggs containing SHR oncospheres without being able to differentiate between *Echinococcus* spp. and *Taenia* spp. eggs [52]. In this study, the number of taeniid eggs used for spiking experiments represents the number of SHR oncospheres. Two taeniid egg suspensions (from two different foxes) were prepared for independent spiking experiments (explained below). The first suspension contained *E. multilocularis* and *Taenia polyacantha* eggs with an SHR rate of 50%. The second egg suspension consisted of *E. multilocularis* eggs only with an SHR rate of 70%. *Toxocara canis* eggs were isolated from worms collected at necropsy as described above. After dissection of female worms, the eggs were embryonated in 0.1 M sulfuric acid [53] and kept at 4 °C in the same suspension until further use. The exact proportion of embryonated eggs was calculated by microscopic examination using a McMaster chamber. Oocysts of *T. gondii* (type II strain CZ clone H3) were isolated from faeces of experimentally infected cats from a study not related to this research (animal experiment ZH040/2017, approved by the Veterinary Office and the Ethics Committee of the Canton of Zurich (Kantonales Veterinäramt Zürich, Zollstrasse 20, 8090 Zürich, Switzerland)). Oocysts were counted using four grids of a Neubauer chamber and the average was calculated to estimate the amount of oocysts in the total volume of suspension.

#### 4.1.2. Spiking of Lettuce with Helminth and Protozoan Parasite Environmental Stages

To assess the efficiency of the strategy presented in this study for the detection of environmental parasite stages, we performed two spiking experiments on lettuce. Specific details of the sieving method and washing of lettuce are described in the next sections. Parasite infective stages (in suspensions of 50–800 µL) were pipetted directly onto three places on the entire lettuce leaves placed inside a polypropylene transparent plastic bag (25.5 × 40.0 cm). In the first experiment (see Table 1), we spiked 300 g of lettuce leaves in five replicates with 20 embryonated *Toxocara* eggs and 20 taeniid eggs (*E*. *multilocularis* and *T. polyacantha*). Another five replicates were spiked with four embryonated *Toxocara* eggs and four taeniid eggs (same as above). As a control, we used 300 g of the same lettuce batch in triplicates which were not spiked. In the second experiment, we spiked 300 g of lettuce leaves in five replicates with 120 eggs of *E. multilocularis* and with 86 *T. gondii* oocysts. Another five replicates were spiked with 40 eggs of *E. multilocularis* and 18 *T. gondii* oocysts. Furthermore, five replicates of 300 g of lettuce spiked with 20, 10 and 2 *E. multilocularis* eggs were also included (see Table 2). Triplicates of 300 g of the same lettuce batch without spiking served as a control. Another three replicates of 300 g of lettuce were spiked with 779 and 139 µL *E*. *multilocularis* egg-free supernatant (after centrifugation, 600× *g*, 15 min) of the spiking suspension (Table 2). This step aimed to assess if free DNA of the parasite is present in the egg suspensions and therefore could be detected with our sieving system.

#### 4.1.3. Sieving System for Isolation of Parasites

**Figure 1:**
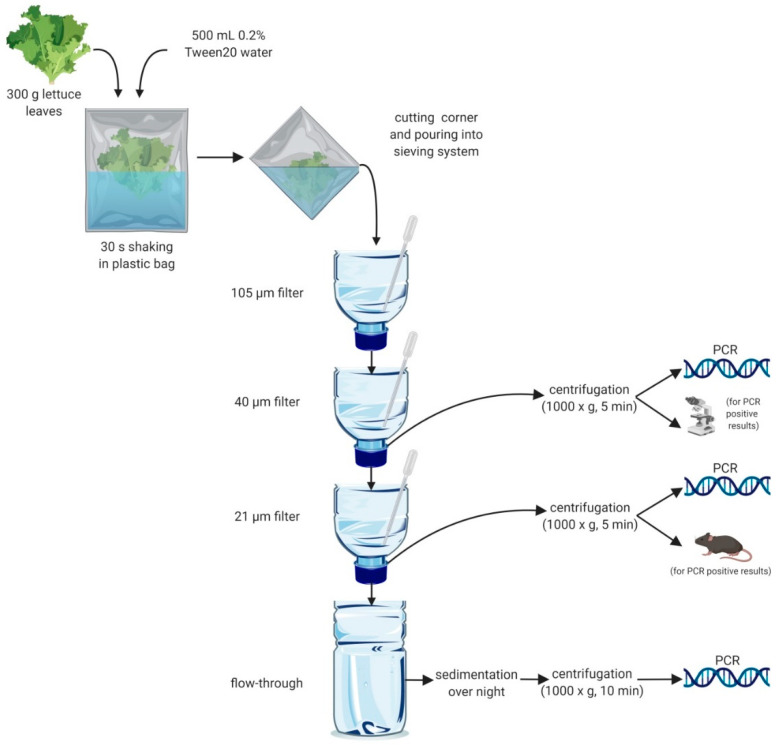
Scheme of the method for washing of lettuce for the detection of taeniid and *Toxocara* eggs and oocysts of *Toxoplasma*. The lettuce leaves were washed by shaking in sealed plastic bags, and the wash water was sieved through a sieving system with 105, 40, and 21 µm mesh size. The material retained on the 40 and 21 µm filters was collected, centrifuged and the pellet divided into two parts. One half was used for PCR, the other for microscopy or the in vivo viability test. The flow-through was sedimented overnight and the pellet analysed by PCR.

A sequential sieving system originally implemented for the detection of taeniids in canine faeces [28] was further developed to isolate a variety of parasite infective stages, based on their size by concentration in nylon filters of different mesh size (105, 40 and 21 µm), or by centrifugation or sedimentation in the flow-through of the system. Used PET bottles of 0.5 and 1.5 L volume were thoroughly washed and cut in half; the top parts were used as funnels and the bottom parts for the collection of the flow-through (Figure 1). The lids of the funnels were perforated, and the different nylon mesh used as filters (Lanz-Anliker AG, Allmendstrasse 12, 4938 Rohrbach, Switzerland) were fixed in the inside of the lid. Funnels were labelled with the corresponding filter size and positioned in a pedestal according to mesh size (top-down 105, 40, 21 µm filter) above the half PET bottle used for the collection of the flow-through (Figure 1). For each sample, a “sieving kit” containing the three different filters and all plastic material was used only once.

#### 4.1.4. Washing of Lettuce

A sample consisting of 300 g of lettuce leaves was placed inside a plastic bag (40 × 26 cm), spiked or not with the parasites mentioned above, and 500 mL of water with 0.2% Tween 20 were added to the bag. The bag was partially closed by twisting the top part to avoid leaking and shaken vigorously for 30 s, holding it with both hands. After 5 min, to reduce bubbles, one corner of the bag was cut (around 5 mm), and the water was carefully poured into the sieving system (Figure 1) while squeezing the bag to get the maximum volume of water. Single-use plastic pipettes facilitated the flow of the liquid in each filter avoiding blockage with dirt (Figure 1). Once all the washing water had passed through the system, 100 mL of water were added on top to thoroughly wash all the filters. The funnels with the 40 µm and 21 µm filters were removed and placed in a small plastic cup containing water to resuspend the filtered material. Using the same plastic pipette, the resuspended material was collected individually and transferred into separate 14 mL tubes. The fluid collected from each filter was thoroughly mixed and divided into two equal aliquots in different tubes which were subsequently centrifuged (1000× *g* for 5 min). After discarding the supernatant, the sediments were transferred to 1.5 mL Eppendorf tubes. From the 40 µm filter, one half was used for molecular detection of *Toxocara*. In the case of a positive result, the second half was used for microscopic detection of eggs in a McMaster chamber after flotation with ZnCl_2_ (density 1.45). From the 21 µm filter, one half was used for PCR for the identification of taeniids, in the case of a positive reaction the other half was used for in vivo assessment of the viability of eggs (Figure 1). The flow-through was concentrated by sedimentation overnight at 4 °C, then the supernatant was discarded, and the sediment collected in a 50 mL Falcon tubes. After centrifugation (1000× *g*, 10 min) the supernatant was removed, and the pellet transferred into 2 mL tubes and stored at −20 °C for molecular detection of *T*. *gondii*. To avoid contamination, all plastic material and filters were used a single time and then discarded.

#### 4.1.5. Molecular Detection of Environmental Stages of *Taeniids*, *Toxocara* and *Toxoplasma*

The material collected from the 21 µm filter in the sieving system was subjected to DNA isolation, as previously described [54]. The material collected in the 40 µm filter was processed similarly, with the slight modification of overnight digestion with proteinase K at 56 °C. For the isolation of DNA from the flow-through, the sediment collected in two tubes was subjected to three steps of freezing/thawing with liquid nitrogen to break the oocyst of *T*. *gondii.* DNA was then isolated from up to one gram of the sediment using the E.Z.N.A.^®^ Soil DNA Kit (Omega Biotek, Norcross, GA, USA) following the manufacturer’s instructions.

DNA isolated from the 21 µm filters was used as a template for a multiplex PCR able to discriminate *E. multilocularis* from other taeniids; importantly the PCR amplicons need to be sequenced as in some cases unspecific bands of similar size could appear when working with environmental samples [26]. DNA isolated from the 40 µm filters was used as a template for classical PCR with primers newly designed using Primer Blast [55] and specificity for *Toxocara* spp. The primers (forward 5′-GGAGTTGTTTAAGTTGGATGG-3′ and reverse 5′-AGAACTCCGCCTTATCAAGACGAC-3′) target the mitochondrial gene *nad2* and part of the tRNA-Ile, producing a 227 bp amplicon. Primers were tested in silico for specificity. For the detection of *T*. *gondii*, DNA isolated from the sediment was subjected to classical PCR targeting the repetitive region B1 as previously described [27]. PCR positive samples were sequenced (Microsynth AG, Balgach, Switzerland) for species confirmation after amplicon purification with the MinElute PCR kit (Qiagen, Hilden, Germany).

### 4.2. Field Study for the Assessment of Parasite Contamination of Lettuce

For testing the practicability of our detecting strategy for infective parasite stages, the presence of taeniid and *Toxocara* eggs and oocysts of *Toxoplasma* on lettuce grown in the surrounding regions of Zürich (Switzerland) was investigated. During summer (June to August) and autumn (September to November) in 2019, lettuce was purchased from local farmer markets and in different supermarkets within the city of Zürich. Different types of lettuce were bought, depending on the daily offer of the vendors, and transferred to the laboratory for processing the same day. The sieving system, which was initially tested using 300 g of lettuce, was upscaled, aiming to examine the highest possible number of lettuce heads as follows. Each diagnostic unit was considered to be a group of nine lettuce heads from which the outer leaves (100 g per head) were taken adding to a total of 900 g. When the nine lettuce heads were heavily contaminated with dirt, the number of leaves examined increased in the magnitude of 300 g up to 1800 g in total. A single “sieving kit” (including all plastic material and filters) was used per diagnostic unit independently of its weight. A total of 157 diagnostic units (approximately 158 kg), corresponding to 1413 lettuce heads, were investigated for the presence of eggs of taeniids and *Toxocara* spp. A subset of 100 of the 157 diagnostic units was also tested for *T*. *gondii*. The molecular detection was performed as described above; viability of infective stages of taeniid and *Toxocara* eggs was performed as described in the next section; no viability testing was performed for *T. gondii*.

### 4.3. Assessment of Viability of Eggs

Egg viability was determined by an in vivo rodent model with few modifications [25]. For eggs of *E*. *multilocularis* from the original egg suspensions as well as from two recovery experiments, five replicates of samples spiked with 2–120 SHR taeniid eggs were used (see results). Furthermore, samples positive for *E*. *multilocularis* eggs in PCR from the field study were also used for assessment of viability. As the material retained on the 21 µm filter from lettuce samples spiked with taeniid eggs or from taeniid PCR positive samples from the field investigation contained a high fraction of dirt, a flotation step with sugar solution (1000× *g*, 30 min) was included prior to the treatment of the eggs with sodium hypochlorite and before injecting them into mice. The final pellet containing the oncospheres was resuspended in 0.2 mL PBS. This suspension was inoculated subcutaneously to a six–eight weeks old female mouse (C57BI/6, purchased at Envigo, Horst, The Netherlands) at the right dorsum behind the forelimb. The animals were monitored up to 12 weeks before necropsy for metacestode growth at the injection site. If this became larger than 1 cm in diameter or if the general condition of the animal was deteriorating, the mouse was euthanised immediately. The ethical permission for the animal trial was given by the Cantonal Veterinary Office of Zürich, Switzerland (permissions 067/16 and 186/19). In the case of *Toxocara* eggs, viability was assessed through microscopic examination by counting the number of embryonated and unembryonated eggs. In the case of *T. gondii*, no in vitro or in vivo viability tests were performed.

### 4.4. Statistical Analysis

Data were analysed using the statistical software IBM SPSS Statistics, Version 26.

## Figures and Tables

**Table 1:** Results of recovery experiment 1 after spiking and washing 300 g of lettuce with different numbers of sodium hypochlorite resistant (SHR) taeniid eggs (*Echinococcus multilocularis* and *Taenia polyacantha*) or embryonated *Toxocara* eggs. PCR results show the proportion of positives out of five or three replicates.

Environmental Stages Used for Spiking					
Embryonated *Toxocara* Eggs			*Echinococcus multilocularis* and *Taenia polyacantha* Eggs Containing Sodium Hypochlorate Resistant (SHR) Oncospheres		
# Eggs	PCR ^1^	Microscopy	# Eggs	PCR ^2^ *E*. *multilocularis*	PCR ^2^ *Taenia*
20	5/5	5/5	20	5/5	2/5
4	5/5	2/5	4	5/5	3/5
0	0/3	0/3	0	0/3	0/3

^1^ Specific detection of *Toxocara* as described in material and methods, ^2^ Trachsel et al. [26].

**Table 2:** Results of recovery experiment 2 after spiking and washing 300 g of lettuce with different numbers of sodium hypochlorite resistant (SHR) eggs of *Echinococcus multilocularis* and sporulated oocysts of *Toxoplasma gondii*. PCR results show the proportion of positives out of five or three replicates.

Environmental Stages Used for Spiking				
*Echinococcus multilocularis* Eggs Containing SHR Oncospheres			*Toxoplasma gondii* Oocysts	
# Eggs (Volume of Spiking Suspension)	PCR ^1^ 1st half	PCR 2nd half	# Oocysts	PCR ^2^
120 (779 µL)	5/5		86	5/5
40 (260 µL)	5/5		18	4/5
20 (130 µL)	5/5		0	0/3
10 (65 µL)	5/5	5/5		
2 ^3^ (20 µL)	5/5	1/5		
0 (none)	0/3	0/3		
0 (779 µL ^4^)	0/3	-		
0 (130 µL ^4^)	0/3	-		

^1^ Trachsel et al. [26], ^2^ Burg et al. [27], ^3^ isolated by microscopy without SHR testing, ^4^ egg-free supernatants of the spiking suspension.

**Table 3:** Detection of taeniid and *Toxocara* DNA from 157 washed lettuce samples bought in farmer markets or supermarkets in the two seasons, summer (June–August) and autumn (September–November).

Location Purchased	Season	Parasite Species	Number of PCR ^1^ Positives
Farmer markets (n = 120)	Summer (n = 57)	*Hydatigera taeniaeformis* (Syn. *Taenia taeniaeformis*)	1
		*T. martis*	1
		*T. polyacantha*	2
		*Toxocara cati*	2
	Autumn (n = 63)	*Echinococcus multilocularis*	2
		*Hydatigera taeniaeformis*	3
		*T. polyacantha*	1
		*Toxocara cati*	1
			Total:13 (10.8%, 95% CI 5.2 to 16.4)
Supermarkets (n = 37)	Summer (n = 23)	*T. cati*	1
	Autumn (n = 14)	-	0
			Total: 1 (2.7%, 95% CI −2.8 to 8.2)

^1^ Trachsel et al. [26] for *Taenia* spp.; for *Toxocara* spp. see materials and methods section. CI: confidence interval.

**Table 4:** Detection of *Toxoplasma gondii* DNA from washed lettuce samples bought in farmer markets or supermarkets in summer (June–August) and autumn (September–November).

Location Purchased	Season	Number of PCR ^1^ Positives
Farmer markets (n = 76)	Summer (n = 15)	1
	Autumn (n = 61)	3
Supermarkets (n = 24)	Summer (n = 10)	0
	Autumn (n = 14)	2

^1^ Burg et al. [27].

**Table 5:** In vivo viability test results of egg inocula and the recovered eggs after spiking and washing 300 g of lettuce with different numbers of sodium hypochlorite resistant (SHR) eggs of mixed *Echinococcus multilocularis* and *Taenia polyacantha* (inoculum 1) and *E. multilocularis* eggs only (inoculum 2).

Egg Suspensions	*Echinococcus multilocularis* and *Taenia polyacantha* Eggs (Inoculum 1)		*E. multilocularis* Eggs (Inoculum 2)	
	# Eggs	In Vivo Development ^1^	# Eggs	In Vivo Development ^1^
Inoculum	40	5/5	60	5/5
	10	2/5	20	2/5
	2	2/5	10	1/5
Recovered after spiking/washing	20	0/5	120	1/5
	4	1/5	40	0/5
	-	-	20	1/5

^1^ PCR of metacestode material positive for *E. multilocularis*.
